# Utility of an Isotonic Beverage on Hydration Status and Cardiovascular Alterations

**DOI:** 10.3390/nu14061286

**Published:** 2022-03-18

**Authors:** Emily E. Bechke, Mitchell E. Zaplatosch, Ji-Yeon Choi, William M. Adams

**Affiliations:** 1 Department of Kinesiology, University of North Carolina at Greensboro, Greensboro, NC 27412, USA; eebechke@uncg.edu (E.E.B.); mezaplat@uncg.edu (M.E.Z.); j_choi7@uncg.edu (J.-Y.C.); 2 Hydration, Environment and Thermal Stress Lab, Greensboro, NC 27412, USA; 3 Division of Sports Medicine, United States Olympic & Paralympic Committee, Colorado Springs, CO 80909, USA

**Keywords:** heart rate variability, systolic time intervals, fluid balance, beverage hydration index, body water, regulation, electrolytes

## Abstract

This study determined the beverage hydration index (BHI) and postprandial cardiac autonomic activity after consuming an isotonic beverage (IB) compared to distilled water (DW). Twenty-two participants (50% female; mean ± SD; age, 27 ± 3 year; height, 169.1 ± 12.6 cm; weight, 73.3 ± 13.8 kg; BF%, 23 ± 10%) completed two experimental trials where they consumed 1 L DW or an IB; after which urine volume and cardiac autonomic activity was measured through 240 min. Cardiac autonomic activity was quantified using heart rate (HR), log transformed heart rate variability measures (root mean square of successive R–R intervals; RMSSD; low frequency, LF; and high frequency, HF) and systolic time intervals (pre-ejection period, PEP). BHI was significantly greater after IB consumption at min 0 (MD [95% CI]; 1.31 [0.35, 2.27]), 180 min (0.09 [0.022, 0.16]), and 240 min (0.1 [0.03, 0.17]) compared to DW (*p* = 0.031). Net fluid balance was significantly greater in IB than DW at 180 min (90 [−16.80, 196.81]) and 240 min (106 [−13.88, 225.88]) (*p* = 0.037). HR decreased over time in both beverage trials but was higher following IB ingestion at 0 min (3.9 [−2.42, 10.22]), 30 min (5.3 [−0.94, 11.54]), and 60 min (2.7 [−3.42, 8.82]) (*p* = 0.0002). lnHF was greater 30 min post DW ingestion compared to IB (0.45 [−0.23, 1.13]) (*p* = 0.039). IB promotes greater fluid retention capacity compared to DW within 4 hours of consumption. The variations in cardiac autonomic measures may warrant further investigation in clinical populations (i.e., patients with autonomic failure).

## 1. Introduction

Alterations in hydration status have been shown to affect both acute and long-term health and performance outcomes such as cardiovascular and thermoregulatory strain, cognitive and physical performance, and increased risk for developing cardiovascular, metabolic, and renal diseases [1,2,3,4]. Inadequate hydration becomes important in situations where access and availability of fluids are restricted (e.g., military operations and remote athletic or occupational settings) or in situations where urination is not desired [5,6,7,8,9]. Thus, commercially available beverages, including sports drinks and oral-rehydration solutions (ORS), have been developed to promote rehydration and fluid retention following activity, but may also promote fluid retention in the absence of acute fluid losses [10]. These beverages typically contain carbohydrates and electrolytes and are formulated to have a concentration that is hypertonic (higher), isotonic (similar), or hypotonic (lower) to body fluids. While the lower osmolality of hypotonic solutions promotes faster intestinal water absorption, the greater energy density of isotonic beverages may promote delayed gastric emptying of fluids to allow for more prolonged maintenance of fluid balance [4].

The desire to understand the hydration potential (i.e., fluid retention capacity) of specific beverages has been explored by Maughan and colleagues wherein the beverage hydration index (BHI) was developed [11]. Findings show that beverages that contain a greater concentration of either macronutrients or electrolytes have a greater potential to retain fluids within the body. A BHI less than 1.0 suggests that the beverage of interest provokes greater urinary excretion than water, whereas a BHI score higher than 1.0 reflects greater fluid retention and suggests additional hydrating properties when compared to water [11,12]. Furthermore, the BHI has shown to be reliable between studies and neither sex, body composition, nor age influence the outcome measure, therefore providing an accurate method across studies to assess hydration properties of various beverages [11,13,14]. 

With the information the BHI provides on fluid retention capacity, it would be beneficial to assess other physiological responses in conjunction with the BHI such as the cardiovascular response. Fluid ingestion is known to cause a number of physiological alterations in the cardiovascular system due to its regulation via the autonomic nervous system. Upon fluid consumption, there is a pressor effect leading to an increase in plasma norepinephrine concentrations leading to vasoconstrictor activity and an acute rise in blood pressure [15,16,17]. In order to counter this effect, healthy individuals release a subsequent discharge of vagal activity to lower heart rate and decrease blood pressure and cardiac output whereas blood pressure significantly increases in those with autonomic dysfunction [16,18,19,20]. Additionally, measures of cardiac autonomic activity have been evaluated following fluid consumption through the use of heart rate variability (HRV). HRV quantifies the timing between consecutive R–R intervals, where greater variability reflects parasympathetic nervous system activity (PNS) and less variability reflects PNS withdrawal [21,22]. PNS activity has shown to increase following fluid consumption in both frequency (e.g., increased high frequency power) and time domain (e.g., increased root mean of successive R–R interval differences) measures [18,19,20,23]. HRV’s ability to capture sympathetic nervous system (SNS) activity is controversial, therefore, pre-ejection period (PEP) calculated from systolic time intervals (STI) has been suggested as a complementary measure of central SNS activity [21,22,24,25,26]. 

However, to the authors’ knowledge, no literature is available that evaluates HRV, STI, and hydration outcomes (i.e., BHI) to an isotonic beverage over a prolonged (240 min) period of time. For those who have limited opportunities to consume fluids, it would be insightful to observe the cardiac autonomic and hemodynamic changes over a longer duration and the effect of different beverages on this response. Thus, the purpose of this study was to (1) determine the fluid retention capacity of an isotonic beverage compared to distilled water and (2) to assess the postprandial cardiac autonomic and hemodynamic alterations following consumption of an isotonic beverage.

## 2. Materials and Methods

### 2.1. Design

**Figure 1:**
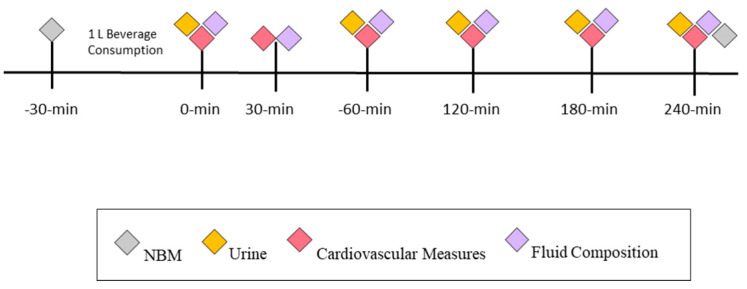
Timeline of measurements. Nude body mass (NBM) was recorded upon arrival followed by the ingestion of 1 L of either distilled water (DW) or isotonic beverage (IB). Either 1 L distilled water (DW) or isotonic beverage (IB) over the course of 30 min. Urine output was measured immediately after fluid consumption and every 60 min for 240 min. Cardiovascular and fluid composition was assessed immediately following, 30 min, and every 60 min following fluid consumption for 240 min.

Utilizing a randomized and crossover design, participants completed two experimental trials where participants consumed 1 L of fluid over a 30-min time period and were then monitored for an additional 240 min. For each experimental trial, participants were randomized to consume either distilled water (DW) or an isotonic rehydration beverage (IB) during their first experimental trial and subsequently the opposite beverage on their second trial. Participants were instructed to record their diet, fluid intake, and exercise for 48 h prior to the first experimental trial and were instructed to replicate their dietary intake and exercise, prior to the second experimental trial (See Figure 1). An a priori power analysis was conducted using G*Power (ver. 3.1.9.7, Dusseldorf, Germany) to determine the number of subjects required for within-between interaction effects. Using an estimated effect size f = 0.25, alpha = 0.05, and power = 0.80 for two groups and five repeated measurements yielded a required sample size of 22 participants. Further, this is in alignment with previous work utilizing the same methodology [14,27,28].

### 2.2. Participants

Male (*n* = 11) and female (*n* = 11) participants between the ages of 18–35 (mean ± SD; age, 27 ± 3 y; height, 169.1 ± 12.6 cm; weight, 73.3 ± 13.8 kg, body fat, 23 ± 10%) volunteered to participate in this study. Following a full explanation of the study protocol, risks, and benefits, volunteers signed an informed consent to participate in this study which was approved by the University of North Carolina at Greensboro’s Institutional Review Board (#20-0101). Prior to participation in the study, participants completed a general health questionnaire to ensure that they did not have evidence of any of the following exclusionary criteria: (1) a clinically relevant disease that may alter body water regulation, (2) previous surgery on the digestive tract that may impair the body’s ability to normally regulate body water, (3) regular drug treatment within the previous 15 days, and (4) actively attempting to gain or lose body weight. To control for consistency between trials, female participants were tested during the follicular (days 1–12) phase of their menstrual cycle to minimize the influence of sex hormones on body water regulation [29,30,31]. Females currently using contraceptives (e.g., IUD) that limit the number of menstrual cycles (≥3) occurring in a given year were excluded from the study to ensure accuracy in the testing periods. Participant characteristics and baseline urine measures can be observed in Table 1. 

### 2.3. Procedures

Following consent, general demographic and anthropometric data were collected. Twenty-four hours before each experimental trial, participants arrived at the laboratory and were provided water to consume over the next 24 h to ensure euhydration and were instructed to void all urine produced over that period into a clean container provided to them to assess 24-h urinary hydration makers. The volume of water provided to each participant to consume met adequate intake recommendations set forth by the European Food Safety Authority (2.0 L for females and 2.5 L for males) [32]. 

On the day of each experimental trial, participants arrived at the laboratory between 0500–0900 h (participants arrived ±1 h between both experimental trials to minimize the effects of circadian variation on body water regulation) following an overnight fast of at least 8 h and refraining from alcohol consumption for 24 h prior to the start of the experimental trial. Upon arrival, participants returned their 24-h urine sample, a nude body mass was recorded, and had electrodes placed on their body for assessment of HRV and STI. 

Following placement of the electrodes, participants were instructed to ingest 1 L of either DW or IB over the course of 30 min (4 equal aliquots of 250 mL every 7.5 min). At the completion of the 30-min time period, participants emptied their bladder and provided cardiovascular and body water composition measures (min 0). Following the 0 min timepoint, body water composition and cardiovascular measures were collected at 30 min (30 min) and then subsequently every 60 min. Participants provided a urine sample at 60-min intervals until a total of 240 min elapsed (0 min, 60 min, 120 min, 180 min, and 240 min) (see Figure 1). During the 60-min urine collection intervals, participants could void their bladder at any time; all urine was collected in a clean container over the course of each 60-min interval and weighed at the respected time point to obtain a measure of urine volume. At the completion of each experimental trial, a final nude body mass was collected.

### 2.4. Beverages and Beverage Preparation

For each experimental trial, participants consumed either 1 L of DW (Deer Park^®^; Nestlé Waters North America Inc., Stamford, CT, USA) or IB (HOIST^®^; QCK, LLC, Cleveland, OH, USA). For IB, the beverage contained the following: 157.720 kcal/L; 30.747 g/L carbohydrate; 909.078 mg/L sodium; and 550.046 mg/L potassium. The volume of each beverage was measured on an electronic scale (Ranger 3000; OHAUS Corporation, Parsippany, NY, USA) to the nearest 0.0001 g and stored at approximately 4–6 °C until time of consumption. Osmolality of the IB was established by averaging three IB from three different cases (281 ± 2 mOsm/L).

### 2.5. Urinary Hydration Measures

From each 24-h urine sample, urine volume (U_VOL_), and urine specific gravity (U_SG_), U_VOL_ was measured on an electronic scale (Ranger 3000; OHAUS Corporation, Parsippany, NY, USA) to the nearest 0.0001 g, where 1 g was assumed to be equal to 1 mL of fluid. U_SG_ was measured using a digital refractometer (Reichert AR200; Reichert Technologies, Buffalo, NY, USA). Throughout the course of the experimental trial, U_VOL_ was measured immediately after fluid consumption (0 min) and every 60 min over the course of 240 min (60 min, 120 min, 180 min, and 240 min). Fluid retention was calculated by measuring the beverage hydration index (BHI) and based on prior work [11]; BHI was calculated by dividing the cumulative urine output of DW by the cumulative urine output of IB.

### 2.6. Anthropometrics and Body Water Compartmentalization

Height was measured to the nearest 0.1 cm using a wall-mounted stadiometer (Model216; Seca, Chino, CA, USA) and nude body mass (NBM) was measured to the nearest 0.1 kg using a digital scale (WB-800S Plus; Tanita Corporation, Tokyo, Japan). For each experimental trial, body water composition (e.g., intra- and extracellular fluid) was measured immediately following fluid consumption (0 min), 30 min post ingestion, and every 60 min via bioelectrical impedance analysis (Quantum IV; RJL Systems, Clinton Township, MI, USA) [33]. Prior to the assessment, participants rested in a supine position for a minimum of 6 min to allow for equilibration of body water compartments (4). Subsequently, electrodes were placed on the ankle, wrist, lower leg, and forearm in order to assess impedance and resistance at 50 KHz at each timepoint. The values for impedance and resistance were further input into the Quantum IV software to calculate intercellular (ICF) and extracellular fluid (ECF). Bioelectrical impendence and the Quantum IV software were also used to assess body fat percentage (BF%) used for subject demographics.

### 2.7. Cardiovascular Measures 

Heart rate (HR) variability (HRV) and systolic time intervals (STI) were collected through a 3-lead electrocardiogram (ECG) and impedance cardiograph (ICG), respectively (MP160^®^; BIOPAC Systems, Inc., Goleta, Ca, USA), using a sampling rate of 2000 Hz. A lead II ECG configuration was placed on the participant which was then later used to analyze HR and HRV. Recordings were collected at 0 min, 30 min, 60 min, 120 min, 180 min, and 240 min while participants were relaxed in the supine position for a total of 6 min and participants were instructed to breathe at a normal respiratory rate. Immediately following this 6 min period, participants remained in the supine position as blood pressure was measured from the right arm.

Heart rate and HRV measures were analyzed during the last 5 mins of each segment, where the first minute was discarded to allow for an adjustment period in order for the cardiovascular system to reach a true resting state. Analysis of HRV and HR measures were completed using Kubios^®^ software version 3.0 (Kubios V 3.0; Joensuu, Finland) where the preprocessing interpolation rate was set to 4 Hz. Each segment was visually inspected by a trained investigator for the presence of artifact or noise. If present, Kubios software was used via the piecewise cubic spline interpolation method to filter the data with a “very low-low artifact correction” and a sensitivity set to identify any R–R abnormalities ±35 s compared to the long local average [34]. However, to avoid misinterpretation of analysis, segments containing three or more irregular R–R intervals were deleted. 

In order to assess PNS activity through the time domain, the root mean square of successive R–R intervals (RMSSD) was chosen for analysis [22]. The Fast Fourier Transformation algorithm was chosen with a set window width of 300 s and 50% overlap to extrapolate data in the frequency domain measures in the low frequency (LF) band (0.04–0.15 Hz), and high frequency (HF) band (0.15–0.4 Hz). Low frequency is a known metric to represent activity from the PNS and SNS, whereas HF represents PNS activity [22].

Thoracic bioimpedance using ICG was used to capture STI, where electrodes were aligned on the neck over the carotid arteries and on the mid-axillary lines. STI was used to calculate PEP which is the most established measure of central sympathetic nervous system activity derived STI [24,35]. PEP reflects the time of ventricular depolarization to the aortic valve opening and was calculated by using the timing between ventricular depolarization (ECG-Q-wave onset) and the opening of the aortic valve (dz/dt B-point) [22,24]. All data were ensemble-averaged over 10 complete cardiac cycles using the data acquisition system software (Acknowledge; BIOPAC Systems, Inc., Goleta, CA, USA).

Blood pressure (BP) was collected using an automatic blood pressure cuff (MOBIL-O-GRAPH^®^; I.E.M. GmbH, Stolberg, Germany). In addition to BP, the following metrics were assessed: mean arterial pressure (MAP) and cardiac output (CO), calculated from measured heart rate and stroke volume (Mobil-O-Graph^®^ Revision 5.1; I.E.M. GmbH, Stolberg, Germany).

### 2.8. Statistical Analysis

All data are presented as mean (SD) or mean difference (MD) (95% confidence intervals), unless otherwise indicated. Welch’s two-sample t-tests were run to compare 24-h urinary hydration markers and nude body mass prior to consuming either test beverage. Separate two-way repeated measures ANOVAs (Drink x Time) were conducted with all urinary variables (BHI, UO, and Net Fluid Balance) and all cardiovascular variables (CO, SBP, DBP, MAP, PEP, HR, RMSSD, HF, and LF), and body water measures (ICV and ECV). All data are presented as means and standard deviations unless otherwise specified. Where significant, Bonferroni post hoc tests were conducted to identify significant pairwise differences between Drink, Time, or Drink x Time interactions. Significance was set at *p* < 0.05, and differences between conditions/time points are presented as mean difference and upper and lower bound 95% confidence intervals. All analyses were conducted using statistical software R with the rstatix package. All HRV metrics (RMSSD, HF, and LF) were log transformed (ln) prior to analysis.

## 3. Results

There were no differences in 24-h urinary hydration status or NBM prior to each experimental trial (*p* > 0.05, Table 1).

### 3.1. Beverage Hydration Index, Net Fluid Balance, and Cumulative Urine Output 

**Figure 2:**
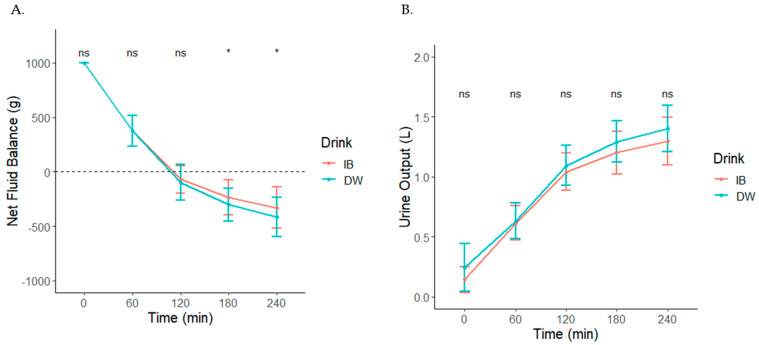
Net fluid balance (**A**) and cumulative urine output (**B**) following consumption of IB or water. * Denotes a significant Drink x Time interaction, *p* < 0.05. Non-significant interactions are denoted by ns.

BHI was greater after IB consumption at 0 min (MD (9.5% CI); 1.31 (0.35, 2.27)), 180 min (0.09 (0.022, 0.16)), and 240 min (0.1 (0.03, 0.17)) compared to DW (*p* = 0.031) (Table 2). Net fluid balance was greater in IB than DW at 180 min (90 (−16.80, 196.81)) and 240 min (106 (−13.88, 225.88)) (*p* = 0.037) (Figure 2A). Cumulative urine output increased over time, regardless of beverage, but there was no Drink x Time interaction for cumulative urine output (*p* = 0.18) (Figure 2B).

### 3.2. Total Body Water, Intracellular Water, and Extracellular Water Measures

Over time, there was a significant decline in TBW (*p* < 0.0001), ICW (*p* = 0.004), and ECW (*p* = 0.004) (Table 2). However, there was no Drink effect or Drink x Time interaction for any BIA measure (*p* > 0.05).

### 3.3. Cardiovascular Measures

**Figure 3:**
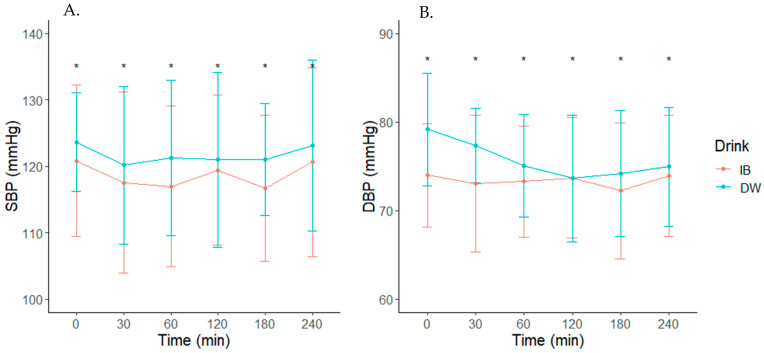
(**A**) Systolic blood pressure (SBP) and (**B**) Diastolic blood pressure (DBP) following consumption of the isotonic beverage (IB) or distilled water (DW). * Denotes a significant main effect of Drink, *p* < 0.05.

**Figure 4:**
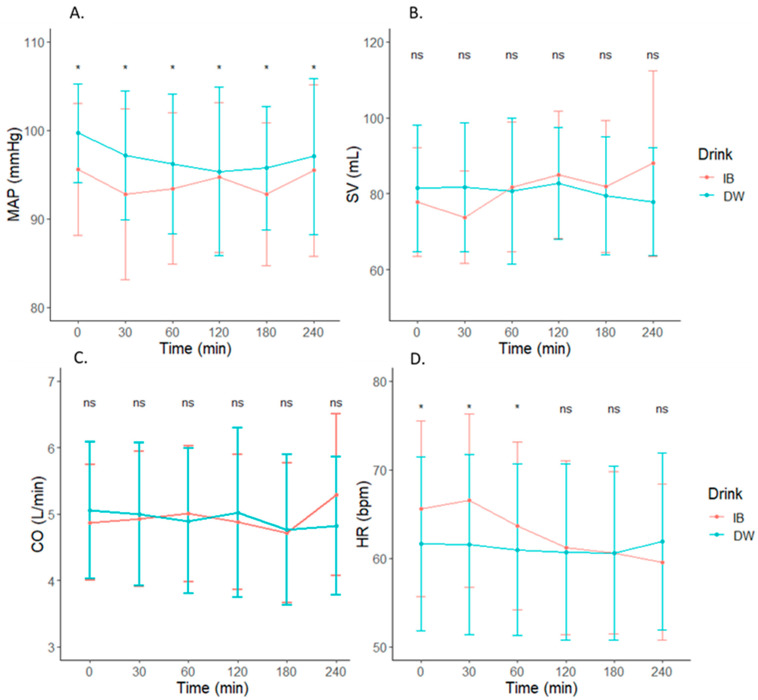
Changes in (**A**) mean arterial pressure (MAP), (**B**) stroke volume (SV), (**C**) cardiac output (CO), and (**D**) heart rate (HR) following consumption of isotonic beverage (IB) or distilled water (DW). * in (**A**) denotes a significant main effect of Drink (*p* < 0.05). * in (**D**) denotes a significant Drink x Time interaction, *p* < 0.05. Non-significant interactions are denoted by ns in all figures.

Raw cardiovascular measures across time are depicted in Table 3. There was no Drink x Time interaction effect for SBP, DBP, or MAP (*p* > 0.05, Figure 3 and Figure 4A). However, there was higher average SBP (3.0 mmHg (0.0440, 5.9560) (*p* = 0.020)), DBP (MD: 2.2 mmHg (0.5196, 3.8804) (*p* = 0.002)), and MAP (MD: 2.5 mmHg (0.4391, 4.5609) (*p* = 0.002)) following DW consumption compared to IB. There was no Drink, Time, or Drink x Time interaction effect for CO or SV (*p* > 0.05) (Figure 4A–C). Mean heart rate decreased over time following either beverage ingestion, but was higher following IB ingestion at 0 (3.9 (−2.42, 10.22)), 30 min (5.3 (−0.94, 11.54)), and 60 min (2.7 (−3.42, 8.82)) post fluid consumption (*p* = 0.0002) (Figure 4D).

**Figure 5:**
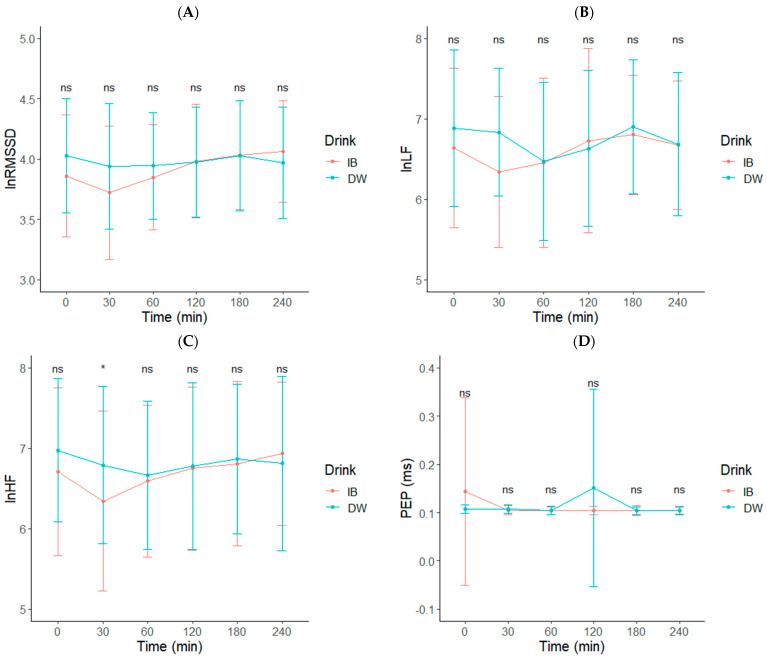
(**A**) Log transformation (ln) of the root mean square (lnRMSSD), (**B**) low frequency (lnLF), (**C**) high frequency (lnHF), and (**D**) pre-ejection period (PEP) following isotonic beverage (IB) or distilled water (DW). * Denotes a significant Drink * Time interaction, *p* < 0.05. Non-significant interactions are denoted by NS in all figures.

There was no Drink x Time interaction for lnRMSSD or lnLF (*p* > 0.05, Figure 5A–D). lnHF was significantly greater 30-min post water ingestion compared to IB. There was no significant Drink (*p* = 0.880), Time (*p* = 0.465), or Drink x Time interaction effect (*p* = 0.371) on the systolic time interval pre-ejection period (PEP) (Figure 5).

## 4. Discussion

The primary findings of the current study show that IB had a BHI greater than DW after 180 min, suggesting greater fluid retention capacity. Similarly, net fluid balance was greater at 180 min and 240 min post ingestion after consuming the IB. However, cumulative urine output over the entire timeframe was not different between the beverages. Our secondary objective was to assess the influence of IB vs. DW on cardiac autonomic measures. Our findings suggest that HR is greater 60 min after consuming an isotonic beverage, while lnHF power was lower 30 min after consuming an IB compared to DW. This may suggest a short-term upregulation in SNS activity following isotonic beverage consumption. Lastly, MAP, SBP, and DBP were higher throughout the trial following DW ingestion vs. IB.

Our findings suggest greater fluid retention capacity was obtained with the IB at 180 min (1.09 ± 0.16) and 240 min (1.1 ± 0.17). The BHI at 60 min (1.05 ± 0.23) and 120 min (1.06 ± 0.15) were not significantly different from DW. The current study’s findings are different from Maughan et al., who found the BHI of an isotonic sports drink to be no different from water at the 120-min time point, although they did not find any differences at 180 min and 240 min post ingestion [11]. The only other study that examined a beverage with a similar osmolality using the BHI was Sollanek et al., who found greater fluid retention (BHI~1.2) 120 min following the ingestion of a glucose oral rehydration solution (osmolality ~267 mmol/kg), which persisted up to 240 min post ingestion [13]. Differences in the timing of these effects may be due to slight variations in beverage composition (i.e., caloric and electrolyte content) and individual subject characteristics (i.e., habitual fluid and electrolyte intake, and glomerular filtration rate). In particular, the beverage used in the present study had a sodium content of 39.9 mmol/L compared to 21 mmol/L in Maughan et al. [11]. Thus, it seems under conditions of euhydration, electrolyte content may have a more profound influence on the fluid retention capacity of a beverage, as has previously been suggested [12,27].

Despite these differences in BHI, bioelectrical impedance measures of ICW, ECW, and TBW remained similar between trials, consistent with observations following isotonic beverage ingestion by Siow et al. [36]. Perhaps differences in fluid retention were the result of unabsorbed fluid remaining in the gut post ingestion, as there were no observed differences in fluid compartments [36]. However, this could also be due to a lack of sensitivity in BIA detecting differences in fluid compartments post fluid ingestion, as has previously been reported [37]. As energy density of beverages increases, the rate of water appearance in the extracellular fluid space decreases as a result of both delays in gastric emptying and intestinal absorption [4,27]. Given the expected movement of fluid to accommodate solute concentration gradients, a slightly hypotonic solution has been suggested to better promote uptake of fluid in the extracellular space by increasing the rate of fluid uptake in the small intestine [4]. However, conditions contributing to an acute increase in serum osmolality (i.e., acute exercise and/or heat stress) may warrant consumption of an isotonic beverage (with respect to normal resting serum osmolality levels in humans) to leverage this return to normal osmolality [4]. Though the IB is advertised as isotonic, the composition could have been slightly hypotonic for some individuals (281 mOsm/kg versus the commonly used 275–290 mOsm/kg reference range), resulting in varied individual absorption and fluid retention rates (see Supplementary Materials Figure S1) (7). Regardless, the IB tested in this study still provided a concentration closer to what would be expected to contribute to an increase in small intestinal fluid uptake. This tonicity may have favored the significantly greater BHI of IB compared to DW, but without measuring participant serum osmolality or gastric or intestinal fluids, we cannot be certain of the mechanism behind this effect.

In terms of cardiovascular and baroreflex buffering parameters, a significant time effect displayed that HR decreased up to 180 min post ingestion of either beverage. HR was significantly higher following IB ingestion up to 60 min post ingestion and had a lower lnHF at 30 min compared to DW. This may suggest PNS withdrawal following IB consumption. Additionally, SBP and DBP were on average 3 mmHg higher following DW consumption, respectively. Previous research investigating cardiac autonomic response following fluid consumption has noted a decrease in HR following water ingestion in a healthy population with little to no change in blood pressure [16,19,20,38]. This decrease in HR following fluid ingestion is primarily observed in healthy young adults as fluid consumption is known to stimulate the SNS and subsequently the PNS to activate greater cardiovagal tone. This theory has been suggested as a means to buffer the pressor effect of fluid ingestion and help regulate blood pressure at a healthy level [15,16]. Lastly, there was no observed time or beverage effect on PEP. To the authors’ knowledge, this is the first study to investigate changes in PEP following fluid consumption as a means to determine SNS activity.

Other research has examined the influence of an isotonic saline solution (0.9%) [19] and commercially available hypertonic solution [39] and has shown a blunted cardiac-autonomic and hemodynamic response when compared to water. Monnard and Grasser observed a similar decrease in HR up to 45 min with no alterations to CO, however they observed an immediate decrease in SBP following water (335 mL) consumption [38]. The extended response of HR in the current study may be due to a dose dependent response considering that our dosage of fluid (1 L) was higher and ingested across a greater time frame (i.e., 30 min) than previous studies [18,40]. Grasser et al. found a prolonged depression in HR following the ingestion of 800 mL of water for up to 60 min when compared to 200, 400, and 600 mL [18]. Additionally, differences between studies may be due to the time allotted for fluid consumption. A majority of the studies examining cardiovascular and cardiac autonomic function had participants drink their fluid at a faster pace (i.e, within 2–10 min), whereas our study had participants consume 250 mL every 7 min for a total of 30 min in order to follow established BHI protocols [11].

At 30 min, the current study showed lnHF was lower when the IB (6.34 ± 1.12) was ingested vs. DW (6.80 ± 0.98) with no significant changes in effect for lnRMSSD between beverages or across time. Additionally, HR was higher following the IB beverage at 0 min, 30 min, and 60 min when compared to DW. Comparably, Christiani et al., observed the cardiac autonomic response of a hypertonic Gatorade solution (591 mL) and water across 60 min and found that there were no significant differences in lnRMSSD, lnSDNN, or R–R intervals between beverages [39]. The observed beverage effects on HR and lnHF are perhaps related to changes in fluid volume or the caloric content of the ingested beverage. In particular, the shift in lnHF may have been due to the carbohydrate consumption, which has been shown to stimulate SNS activity via increases in norepinephrine [41,42]. The total carbohydrate intake from 1 L of the IB was 30.747 g/L, a modest amount compared to studies observing the effects of high carbohydrate meals on cardiac autonomic activity. These findings may be the result of an interaction between fluid regulatory hormones and cardiac autonomic modulation. Acute increases in atrial natriuretic hormone (ANP) occur in response to an expansion of the extracellular fluid space [43]. In turn, increased ANP has also been associated with elevated HF and a decreased LF/HF ratio among older adults [44]. The differences between beverages on HR and lnHF in the present study could theoretically have been influenced by the rapid decline in serum osmolality following acute water ingestion, promoting greater urine output in synergy with the actions of ANP [45]. However, assessing changes in ANP and serum osmolality were beyond the scope of the present study.

While interpreting the findings of the current study, it is important to note the current study design differed from others as it did not include a baseline measure of cardiovascular parameters prior to fluid consumption [19,23,38,39,40]. Instead, the first measurement of cardiovascular and fluid composition was obtained immediately following fluid ingestion. This study design choice was because the primary outcome was to evaluate alterations in fluid balance. Additionally, cardiovascular measures were compared to the DW group to evaluate differences between beverages. However, this component of our study design is recognized as a flaw and future research should include a baseline metric prior to fluid consumption. For instance, although BP was higher during the water trial, it is hard to determine if this response is related to the beverage or stress levels of the participants without a baseline measure prior to fluid consumption. Additionally, it would have been ideal to have a control condition (i.e., a trial without fluid consumption) to determine the magnitude of the alterations we observed.

## 5. Conclusions

In conclusion, our findings suggest an IB promotes greater fluid retention capacity compared to DW up to 240 min. The variations in cardiovascular and cardiac autonomic measures may warrant further investigation in clinical populations (i.e., patients with autonomic failure), given the observed increase in HR and decrease in lnHF in the early post ingestion period.

## Figures and Tables

**Table 1:** Participant Demographic Characteristics. Values are presented as means (±SD).

	IB (N = 22)	DW (N = 22)	*p*-Value
Height (cm)			
Mean (SD)	169.1 (±12.59)	169.1 (±12.59)	
Body Mass (kg)			
Mean (SD)	73.27 (±13.83)	73.27 (±13.83)	
Age (years)			
Mean (SD)	27.1 (±3.5)	27.1 (±3.5)	
Sex			
Female	11 (50%)	11 (50%)	
Male	11 (50%)	11 (50%)	
Arrival U_SG_			
Mean (SD)	1.016 (±0.008)	1.013 (±0.009)	0.1879
Missing	0 (0%)	2 (9.1%)	
24h U_SG_			
Mean (SD)	1.013 (±0.006)	1.013 (±0.005)	0.8655
24h Urine Volume (L)			
Mean (SD)	2.128 (±1.040)	2.153 (±1.161)	0.9402
Arrival Body Mass (kg)			
Mean (SD)	73.30 (±14.14)	73.68 (±14.34)	0.9312

IB = Isotonic Beverage; DW = Distilled Water; U_SG_ = Urine Specific Gravity; SD = Standard Deviation.

**Table 2:** Differences in hydration biomarkers throughout the 240 min post ingestion of either isotonic beverage (IB) or distilled water (DW). * = *p* < 0.05.

	60 (min)		120 (min)		180 (min)		240 (min)	
	IB	DW	IB	DW	IB	DW	IB	DW
	(N = 22)	(N = 22)	(N = 22)	(N = 22)	(N = 22)	(N = 22)	(N = 22)	(N = 22)
BHI			00A0					
Mean (SD)	1.05 (±0.23)	1.00 (±0)	1.06 (±0.15)	1.00 (±0)	1.09 (±0.16) *	1.00 (±0)	1.10 (±0.17) *	1.00 (±0)
Urine Output (L)								
Mean (SD)	0.6157 (±0.1428)	0.6327 (±0.1478)	1.043 (±0.1528)	1.094 (±0.1678)	1.202 (±0.1788)	1.292 (±0.1724)	1.299 (±0.1995)	1.405 (±0.1939)
TBW (L)								
Mean (SD)	37.59 (±8.43)	37.19 (±7.77)	37.54 (±8.22)	37.02 (±7.65)	37.37 (±8.20)	36.93 (±7.48)	37.30 (±8.25)	36.79 (±7.64)
Missing	5 (22.7%)	5 (22.7%)	5 (22.7%)	5 (22.7%)	5 (22.7%)	5 (22.7%)	5 (22.7%)	5 (22.7%)
ICW (L)								
Mean (SD)	20.88 (±5.27)	20.69 (±4.98)	20.85 (±5.19)	20.60 (±4.90)	20.79 (±5.18)	20.59 (±4.78)	20.76 (±5.20)	20.51 (±4.89)
Missing	5 (22.7%)	5 (22.7%)	5 (22.7%)	5 (22.7%)	5 (22.7%)	5 (22.7%)	5 (22.7%)	5 (22.7%)
ECW (L)								
Mean (SD)	16.71 (±3.47)	16.49 (±3.11)	16.69 (±3.35)	16.42 (±3.08)	16.58 (±3.33)	16.34 (±3.04)	16.54 (±3.37)	16.28 (±3.08)
Missing	5 (22.7%)	5 (22.7%)	5 (22.7%)	5 (22.7%)	5 (22.7%)	5 (22.7%)	5 (22.7%)	5 (22.7%)

BHI = Beverage Hydration Index; TBW = Total Body Water; ICW = Intracellular Water; ECW = Extracellular Water.

**Table 3:** Differences in cardiovascular and cardiac autonomic measures throughout the 240 min post ingestion of either the isotonic beverage (IB) or distilled water (DW). * Denotes a significant Drink x Time interaction, *p* < 0.05.

	0 (min)		30 (min)		60 (min)		120 (min)		180 (min)		240 (min)	
	IB	DW	IB	DW	IB	DW	IB	DW	IB	DW	IB	DW
	(N = 22)	(N = 22)	(N = 22)	(N = 22)	(N = 22)	(N = 22)	(N = 22)	(N = 22)	(N = 22)	(N = 22)	(N = 22)	(N = 22)
CO (L/min)												
Mean (SD)	4.871 (±0.8707)	5.061 (±1.028)	4.929 (±1.020)	5.000 (±1.078)	5.005 (±1.028)	4.895 (±1.094)	4.886 (±1.017)	5.025 (±1.275)	4.719 (±1.055)	4.763 (±1.138)	5.289 (±1.216)	4.825 (±1.040)
Missing	1 (4.5%)	4 (18.2%)	5 (22.7%)	7 (31.8%)	2 (9.1%)	2 (9.1%)	1 (4.5%)	2 (9.1%)	1 (4.5%)	3 (13.6%)	3 (13.6%)	2 (9.1%)
SV (mL/beat)												
Mean (SD)	80.36 (±14.64)	85.36 (±21.09)	77.44 (±16.52)	83.04 (±16.16)	85.32 (±17.47)	84.99 (±22.60)	87.57 (±17.31)	87.78 (±22.56)	84.00 (±16.77)	81.78 (±18.91)	93.79 (±26.46)	82.16 (±17.11)
Missing	1 (4.5%)	4 (18.2%)	5 (22.7%)	7 (31.8%)	2 (9.1%)	2 (9.1%)	1 (4.5%)	2 (9.1%)	1 (4.5%)	3 (13.6%)	3 (13.6%)	2 (9.1%)
MAP (mmHg)												
Mean (SD)	95.33 (±7.351)	99.40 (±5.567)	93.67 (±10.02)	96.59 (±7.467)	93.24 (±8.396)	96.00 (±7.760)	94.67 (±8.272)	95.25 (±9.262)	92.52 (±7.960)	95.65 (±6.784)	95.16 (±9.553)	96.95 (±8.593)
Missing	1 (4.5%)	2 (9.1%)	4 (18.2%)	5 (22.7%)	1 (4.5%)	2 (9.1%)	1 (4.5%)	2 (9.1%)	1 (4.5%)	2 (9.1%)	3 (13.6%)	2 (9.1%)
Systole (mmHg)												
Mean (SD)	120.9 (±11.40)	123.7 (±7.464)	117.6 (±13.63)	120.2 (±11.88)	117.0 (±12.16)	121.3 (±11.71)	119.4 (±11.30)	121.0 (±13.15)	116.7 (±11.02)	121.1 (±8.457)	120.6 (±14.26)	123.2 (±12.91)
Missing	1 (4.5%)	2 (9.1%)	4 (18.2%)	5 (22.7%)	1 (4.5%)	2 (9.1%)	1 (4.5%)	2 (9.1%)	1 (4.5%)	2 (9.1%)	3 (13.6%)	2 (9.1%)
Diastole (mmHg)												
Mean (SD)	73.71 (±5.824)	78.85 (±6.352)	73.61 (±7.875)	76.82 (±4.653)	73.24 (±6.107)	74.70 (±5.850)	73.62 (±6.614)	73.55 (±6.932)	71.95 (±7.619)	74.25 (±6.950)	73.63 (±6.809)	74.75 (±6.632)
Missing	1 (4.5%)	2 (9.1%)	4 (18.2%)	5 (22.7%)	1 (4.5%)	2 (9.1%)	1 (4.5%)	2 (9.1%)	1 (4.5%)	2 (9.1%)	3 (13.6%)	2 (9.1%)
lnRMSSD												
Mean (SD)	3.862 (±0.5067)	4.029 (±0.4758)	3.723 (±0.5523)	3.938 (±0.5201)	3.848 (±0.4349)	3.945 (±0.4407)	3.984 (±0.4683)	3.974 (±0.4568)	4.035 (±0.4520)	4.029 (±0.4570)	4.063 (±0.4230)	3.967 (±0.4618)
Missing	2 (9.1%)	2 (9.1%)	2 (9.1%)	3 (13.6%)	2 (9.1%)	2 (9.1%)	3 (13.6%)	2 (9.1%)	3 (13.6%)	2 (9.1%)	4 (18.2%)	2 (9.1%)
lnHF												
Mean (SD)	6.710 (±1.047)	6.977 (±0.8894)	6.342 (±1.119) *	6.793 (±0.9807)	6.592 (±0.9440)	6.666 (±0.9251)	6.753 (±1.010)	6.775 (±1.042)	6.808 (±1.021)	6.866 (±0.9305)	6.936 (±0.8913)	6.812 (±1.081)
Missing	2 (9.1%)	2 (9.1%)	2 (9.1%)	3 (13.6%)	2 (9.1%)	2 (9.1%)	3 (13.6%)	2 (9.1%)	3 (13.6%)	2 (9.1%)	4 (18.2%)	2 (9.1%)
lnLF												
Mean (SD)	6.639 (±0.9908)	6.883 (±0.9713)	6.343 (±0.9375)	6.837 (±0.7925)	6.454 (±1.055)	6.473 (±0.9820)	6.730 (±1.145)	6.632 (±0.9689)	6.802 (±0.7407)	6.902 (±0.8313)	6.675 (±0.7989)	6.685 (±0.8903)
Missing	2 (9.1%)	2 (9.1%)	2 (9.1%)	3 (13.6%)	2 (9.1%)	2 (9.1%)	3 (13.6%)	2 (9.1%)	3 (13.6%)	2 (9.1%)	4 (18.2%)	2 (9.1%)

CO = cardiac output; SV = Stroke Volume; MAP = Mean Arterial Pressure; lnRMSSD = log-transformed Root Mean Square of Successive Differences, lnHF = log-transformed High Frequency Power; lnLF = log-transformed Low Frequency Power.

## Data Availability

Data are available upon request to the corresponding author.

## Supplementary Materials

The following are available online at https://www.mdpi.com/article/10.3390/nu14061286/s1, Figure S1: Individual participant changes in the Beverage Hydration Index (BHI) following ingestion of an isotonic beverage (IB) compared to distilled water at timepoints 60 min, 120 min, 180 min, and 240 min. BHI of 0 denotes no difference in fluid retention compared to water.
