# Supplementary material for: Utility of an Isotonic Beverage on Hydration Status and Cardiovascular Alterations
Source: Nutrients. 2022 Mar 18;14(6):1286. doi: 10.3390/nu14061286 (PMC8953172; doi:10.3390/nu14061286)
Supplement: Supplementary file 1 [file nutrients-14-01286-s001.zip › nutrients-1628058-supplementary.pdf]

Supplementary Materials S1

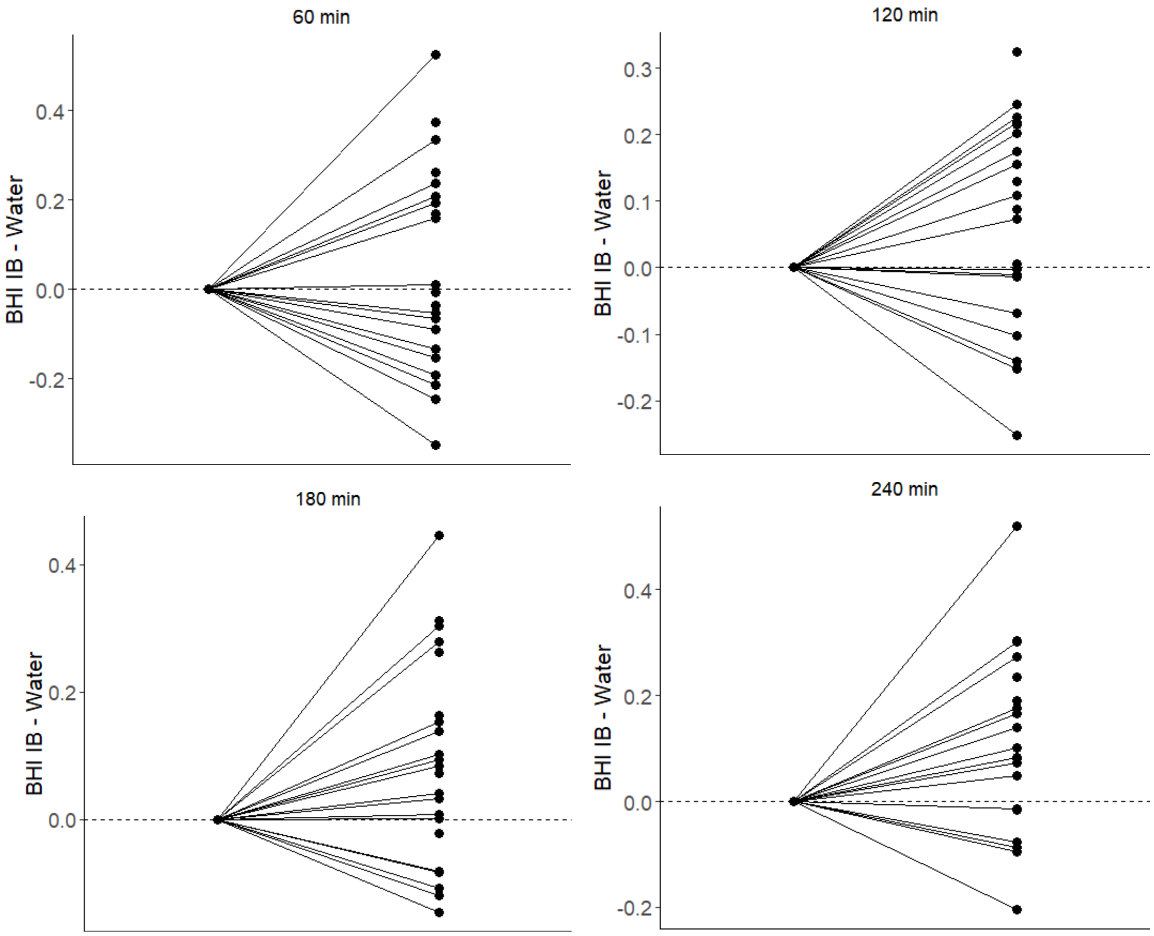

Figure S1. Individual participant changes in the Beverage Hydration Index (BHI) following ingestion of an isotonic beverage (IB) compared to distilled water at timepoints 60 min, 120 min, 180 min, and 240 min. BHI of 0 denotes no difference in fluid retention compared to water.
